# Traumatic Optic Nerve Injury Elevates Plasma Biomarkers of Traumatic Brain Injury in a Porcine Model

**DOI:** 10.1089/neu.2020.7039

**Published:** 2021-04-05

**Authors:** Gregory T. Bramblett, Jason N. Harris, Laura L. Scott, Andrew W. Holt

**Affiliations:** United States Army Institute of Surgical Research, Fort Sam Houston, Texas, USA.

**Keywords:** optic nerve, porcine model, traumatic optic neuropathy

## Abstract

A diagnosis of traumatic brain injury (TBI) is typically based on patient medical history, a clinical examination, and imaging tests. Elevated plasma levels of glial fibrillary acidic protein (GFAP), ubiquitin c-terminal hydrolase L1 (UCH-L1), and neurofilament light chain (NFL) have been observed in numerous studies of TBI patients. It is reasonable to view traumatic optic neuropathy (TON) as a focal form of TBI. The purpose of this study was to assess if circulating GFAP, UCH-L1, and NFL are also elevated in a porcine model of TON. Serum levels of GFAP, UCH-L1, and NFL were measured immediately before optic nerve crush and 1 h post-injury in 10 Yucatan minipigs. Severity of optic nerve crush was confirmed by visual inspection of the optic nerve at time of injury, loss of visual function as measured by flash visual evoked potential (fVEP) at 7 and 14 days, and histological analysis of axonal transport of cholera toxin-β (CT-β) within the optic nerve. Post-crush concentrations of GFAP, UCH-L1, and NFL were all significantly elevated compared with pre-crush concentrations (*p* < 0.01, *p* = 0.01, and *p* < 0.01, respectively). The largest increase was observed for GFAP with the post-injury median concentration increasing nearly sevenfold. The use of these TBI biomarkers for diagnosing and managing TON may be helpful for non-ophthalmologists in particular in diagnosing this condition. In addition, the potential utility of these biomarkers for diagnosing other optic nerve and/or retinal pathologies should be evaluated.

## Introduction

A diagnosis of traumatic brain injury (TBI) is based on patient medical history, a clinical examination, and imaging tests.^1–5^ The ability to diagnose and classify TBI as well as to decide when imaging is indicated has advanced with the identification of circulating biomarkers often elevated in TBI patients. Specifically, the combined measurement of glial fibrillary acidic protein (GFAP, an abundant intermediate filament contained within astrocytes) and ubiquitin c-terminal hydrolase L1 (UCH-L1, a neuronal cystosolic enzyme) was recently approved by the United States Food and Drug Administration (FDA) as part of the Banyan Trauma Indicator (BTI) to aid in the evaluation of patients ≥18 years of age with a suspected TBI (Glasgow Coma Scale [GCS] score 13–15) when collected within 12 h of the injury. ALERT-TBI, the landmark multi-center observational study, key in obtaining FDA approval for these biomarkers, collected data from 1959 patients with suspected non-penetrating TBI and a Glasgow Coma Scale score of 9–15. The authors found that the combined use of GFAP and UCH-L1 had a sensitivity of 97.6% and negative predictive value (NPV) of 99.6% for the detection of acute intracranial lesions on computed tomography (CT).^6^ Other biomarkers, including neurofilament light chain (NFL, a neuron specific intermediate filament) are also elevated in TBI but are not FDA approved for use in TBI evaluation.^6–20^

It is reasonable to consider isolated traumatic optic neuropathy (TON) a focal form of TBI. The optic nerve is a specialized structure of the central nervous system (CNS) that serves the visual system. Although it is the second member of the 12 cranial “nerves,” the optic nerve is actually a white matter tract of the brain. It is (1) embryologically derived from the diencephalon; (2) myelinated by oligodendrocytes rather than Schwann cells; (3) surrounded by layers of dura, arachnoid, and pia as elsewhere in the brain; and (4) has a blood–brain barrier.^21–30^ Its axons are derived from retinal ganglion cells (RGCs), which are themselves specialized CNS neurons.^27,31^ Like other CNS neurons, damage to these axons results in Wallerian degeneration and apoptosis of their RGC cell bodies.^32,33^

As the name would suggest, TON is an injury caused by trauma to the optic nerve. TON injuries are usually divided into either direct (e.g., penetrating injury) or indirect mechanisms of injury (e.g., concussive force transmitted to the optic nerve with globe or head trauma). Visual loss is usually immediate, but can be delayed in at least 10% of cases, presumably via secondary mechanisms that have not yet been fully elucidated.^34^ A prospective surveillance study conducted in the United Kingdom found that TON affects 1 in 1,000,000 of the general population.^35^ TON is present in 0.7–2.5% of cases of blunt and penetrating head and facial injury. Younger adult males predominate and male prevalence and other characteristics of TON are similar in pediatric and adult populations.^36,37^ In a combat environment, the incidence of eye injuries among all combat-related injuries has been reported as being 13%, with TON making up 20% of eye-related injuries.^38^ Interestingly, seemingly trivial injuries such as trauma to the forehead with no alteration of awareness or loss of consciousness can cause TON.^39^ However, the trauma associated with TON is more often severe. Prospective and retrospective studies show that 34–75% of TON cases are associated with a loss of consciousness. Affected eyes have no light perception 36–82% of the time.^35,40,41^ There have not been any well-designed prospective studies to delineate optimum clinical management of patients with TON. Current mainstay management options are steroids, surgical management, and/or observation.^34^

As with TBI, a TON diagnosis is based on patient medical history and clinical examination. In TON, imaging and other tests serve an adjunct role. Obtaining a medical history and testing for a relative afferent pupillary defect (RAPD, the “swinging flashlight” examination), visual acuity, color plate testing, the dilated fundoscopic examination (DFE) and static optic perimetry are mainstays of establishing a TON clinical diagnosis. Optical coherence tomography (OCT), magnetic resonance imaging (MRI), visual evoked potentials (VEP), CT, and ultrasound are all additional adjunctive tools.^34^

Although all of these methods can be very useful, they have some inherent limitations. For example, testing for an RAPD is often limited by the examiner's experience. This is problematic, as testing for an RAPD is considered by neuro-ophthalmologists to be the most important clinical test in diagnosing TON. Non-ophthalmologists in particular often fail to properly perform and interpret this test. Imaging and ancillary tests such as OCT will also frequently be unrevealing, especially in the acute stages of disease, as it can take days to even weeks for some of these tests to show evidence of injury, if they do at all.^34^ Currently, no laboratory test exists to specifically assess the structural integrity or function of the optic nerve in a traumatic setting.

The purpose of this study was to assess if the biomarkers elevated after TBI were also elevated after development of isolated TON in a porcine model. This was a small nested study, conducted as part of a larger experiment investigating potential optic nerve regeneration, the details of which will be described in future publications. The Quanterix Simoa Human Neurology 4-plex A (N4PA) kit, which targets GFAP, UCH-L1, NFL, and total tau (t-tau, a group of alternatively spliced, axon-resident, microtubule-associated phosphoproteins), was used to measure these biomarkers in plasma. This assay is not FDA approved for clinical use, but is currently being used across the world for research purposes.

In efforts to elucidate the conditions conducive to the regeneration of RGCs and their axons, a well-established murine optic nerve crush model of TON has been developed, characterized, and extensively used.^42–44^ Using this model as a paradigm, we developed a porcine optic nerve crush model of TON to research neuroprotective and neuro-regenerative optic nerve treatments that have had some success in the murine model.^45^ Our porcine TON model demonstrates loss of visual function as proven by pathophysiological depression of flash visual evoked potential (fVEP) in addition to loss of normal physiological function of the optic nerve as demonstrated by abolished anterograde, axonal transport of cholera toxin-β subunit (CT-β).^46^ Within this investigation of optic nerve regeneration, we performed this pilot study to determine if the TBI biomarkers mentioned are also elevated in isolated TON.

## Methods

Ten female Yucatan minipigs each weighing ∼9–13 kg were independently evaluated and screened for pre-existing disease 3–7 days prior to the surgical procedure. Baseline flash fVEP were obtained at this time with a RETeval handheld electrophysiology device (LKC Technologies; Gaithersburg, MD) to ensure objective evidence of visual function. Access to the orbital portion of the optic nerve was achieved by lateral orbitotomy. Once generous exposure of the optic nerve was obtained (mean post-initial anesthesia time ± standard deviation (SD): 5 h 21 min ±49 min), blood was drawn from the right saphenous vein using standard phlebotomy techniques. A 23 gauge butterfly needle and lavender top tubes were used to collect two mL of whole blood immediately before and then 1 h after traumatic optic nerve injury. Injury was performed by crushing the optic nerve with a neurosurgical aneurysm clip (Aesculap Inc., Center Valley, PA, CAT No. FT820T) and reinforcing it by the addition of a titanium reinforcing clip (Aesculap Inc., Center Valley, PA, CAT No. FT900T), inducing ∼12 N of crush force at the application site (measured using FlexiForce ELF System [Tekscan; South Boston, MA]). Blood was centrifuged at 2013*g* for 20 min at 4°C, and plasma volumes were aliquoted into 1.5 mL tubes and stored at -80°C. Plasma samples were shipped overnight to Quanterix (Billerica, MA) on dry ice. The Simoa Human Neurology 4-plex A (N4PA) assay was performed by Quanterix personnel under blinded conditions. Samples were run in duplicate at a dilution factor of four. Appropriate plate controls to establish limits of detection (LOD) and limits of quantification (LOQ) were used.

To confirm the validity and reproducibility of the crush lesions and that loss of visual function and loss of normal physiological measures had occurred, optic nerve crush was clearly visualized in each animal by the surgeon. Weekly serial fVEPs of the right and left eyes were obtained with the animals under general anesthesia. Axonal transport within the optic nerves using CT-β was also assessed. Two days before study end-point, 200 μg of CT-β in 150 μL of sterile saline was injected into the vitreous cavity of the left (uncrushed), and right (crushed) eyes of each pig. Eyes were fixed by transcardial perfusion fixation with 10% neutral buffered formalin with the animals under general anesthesia. The eyes and optic nerves were harvested, and then the optic nerves and peripapillary retina were prepped for frozen sectioning.

Antibodies in the N4PA assay are known to cross-react with three of the four biomarkers from porcine samples. However, the monoclonal antibody used in the assay to measure the t-tau biomarker in human plasma does not cross-react with porcine t-tau (see Quanterix application note “Porcine Cross Reactivity in Human Simoa Assays”). Therefore, the t-tau biomarker data were not included in the statistical analysis. Samples with biomarker concentrations below the LOQ were assumed to have a concentration equal to the biomarker-specific LOQ (*n* = 4).

### Statistical analysis

Differences between pre- and post-injury concentrations were examined using the Wilcoxon signed rank test. All analyses were conducted using SAS version 9.4 (Cary, NC), and statistical significance was evaluated using an α of 0.05.

## Results

**FIG. 1.**
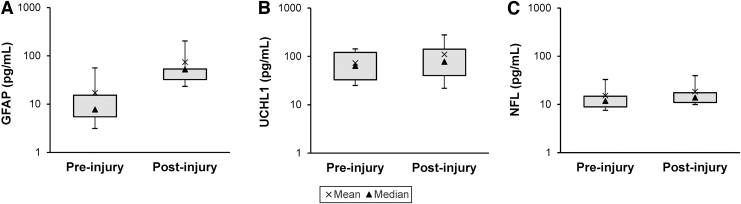
Distribution of plasma concentrations of traumatic brain injury (TBI) biomarkers before and after optic nerve (ON) crush. Post-injury concentrations of all biomarkers were significantly elevated compared with pre-injury concentrations. Whiskers represent the 10th and 90th percentiles. **(A)** Glial fibrillary acidic protein (GFAP): pre-injury median (interquartile range [IQR]) concentration is 7.76 pg/mL (5.48–15.3) and post-injury median (IQR) concentration is 52.3 pg/mL (32.3–53.4) (*p* < 0.01). **(B)** Ubiquitin c-terminal hydrolase L1 (UCH-L1): pre-injury median (IQR) concentration is 63.6 pg/mL (32.8–121.0) and post-injury median (IQR) concentration is 77.6 pg/mL (40.1–141.0) (*p* = 0.01). **(C)** Neurofilament light chain (NFL): pre-injury median (IQR) concentration is 11.8 pg/mL (8.89–14.8) and post-injury median (IQR) concentration is 13.9 pg/mL (11.0–17.5) (*p* < 0.01).

The distributions of pre- and post-injury biomarker concentrations for GFAP, UCH-L1, and NFL are illustrated in Figure 1. All three were significantly elevated post-crush. The largest increase was observed for GFAP, with a pre-injury median (interquartile range [IQR]) concentration of 7.76 pg/mL (5.48–15.3) and a post-injury median (IQR) concentration of 52.3 pg/mL (32.3–53.4) (*p* < 0.01). Increases in UCH-L1 (pre-injury median [IQR]: 63.6 pg/mL [32.8-121.0] vs. post-injury median [IQR]: 77.6 pg/mL [40.1–141.0]) and NFL (pre-injury median [IQR]: 11.8 pg/mL [8.89-14.8] vs. post-injury median [IQR]: 13.9 pg/mL [11.0-17.5]) were much smaller, but still statistically significant (*p* = 0.01 and *p* < 0.01, respectively).

**FIG. 2.**
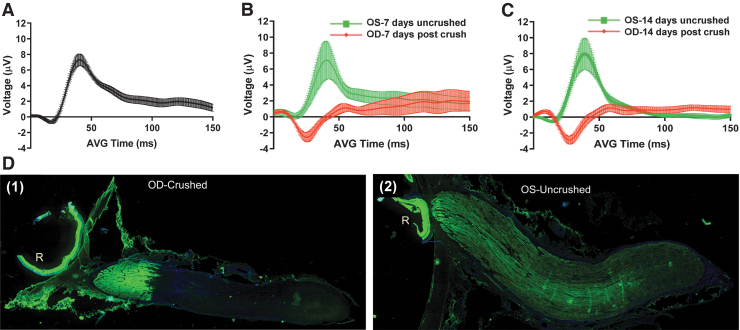
Assessment of porcine optic nerve form and function before and after optic nerve crush. **(A)** Baseline flash visual evoked potential (fVEP) recordings were obtained from 15 Yucatan minipigs 3–7 days prior to optic nerve crush. For all fVEP recordings, replicates were performed for oculus dextrus (OD) followed by oculus sinister (OS) (*n* = 30 waveforms). fVEP recording 7 days **(B)** and 14 days **(C)** after optic nerve crush from saline-only injected minipigs (*n* = 4 pigs, 8 waveforms per eye/per time point). **(D)** Axonal transport of cholera toxin-β subunit (CT-β) in uncrushed and crushed optic nerves. Two days before euthanasia and harvest of eyes and optic nerves, 500 μg of CT-β was dissolved in 250 μL of injectable saline and 100 μL of CT-β solution was injected into the vitreous of both eyes. Two days after injection, the pigs were euthanized via transcardial perfusion fixation with 10% formalin, and the eyes and optic nerves were harvested. The optic nerves and peripapillary retina were then prepared for frozen sectioning. The right (crushed) optic nerve **(D1)** shows fluorescent signal from the CT-β up to, but not beyond, the crush site. In the immunofluorescence pattern obtained from the left eye **(D2)**, the CT-β signal can be seen through the entire length of the nerve, demonstrating intact, unimpaired axonal transport throughout its entire length.

There was strong evidence the optic nerves were severely and traumatically injured. Severe crush was visually confirmed at the time of crush by the surgeon. Normal fVEPs were evident in uncrushed control eyes compared with a remarkably flat fVEP (after N1 waveform) in crushed eyes (Fig. 2, A–C). The flat fVEP response, indicating loss of visual function, fully appeared 7 days after optic nerve crush. This response was maintained through 14 days post-injury (Fig. 2 B and C). Additionally, axonal transport of CT-β was almost completely abolished posterior to the crush site (Fig. 2 D).

## Discussion

The diagnosis and management of many diseases has benefited from the revolution in molecular diagnostics. However, this largely has not been the case in the diagnosis of optic nerve pathology. TON is a significant cause of trauma-related vision loss. Using a plasma-based enzyme-linked immunosorbent assay to measure biomarker concentrations in a porcine model of TON, we observed significant increases in the TBI biomarkers GFAP, UCH-L1, and NFL, with the post-injury median concentration of GFAP increasing nearly sevenfold. This is the first documented instance of TON being studied or detected using a laboratory assay of plasma biomarkers.

The research presented here is subject to a number of limitations. A relatively small number of animals (*n* = 10) was used. Post-hoc power calculations revealed that analyses of both the GFAP and NFL data were sufficiently powered (1-β > 80%). Statistical analysis of UCH-L1 data was underpowered, likely as a result of the large variability in the post-injury concentrations. Nonetheless, differences in pre- and post-injury concentrations were statistically and clinically significant for all three biomarkers. Another limitation of the current study was that only female pigs were used. Although we were unable to evaluate whether biomarker concentrations varied by sex, exclusion of male pigs was also a strength of the study as it prevented any potential confounding because of this factor. Finally, the severity of the optic nerve crush in this animal model is a weakness in that the nature of the injury is much more severe than many clinical cases of TON. Indeed, there was no doubt that each animal's optic nerve was traumatically crushed. However, the severity of the injury prevented us from evaluating whether these biomarkers would be elevated in milder cases of TON.

As described previously, the broader objective of the primary experiment was to investigate porcine optic nerve regeneration. To measure TBI biomarkers as part of a sub-experiment, plasma samples were collected immediately before the optic nerve was crushed and 1 h post-crush. The 1 h time point post-crush was chosen for two reasons. First, previous work using a swine model of TBI showed a linear increase in circulating GFAP and NFL concentrations 2 h after injury (see Quanterix application note “Porcine Cross Reactivity in Human Simoa Assays”), indicating that biomarker concentrations would also likely increase at 1 h post-injury. Second, because treatments to promote optic nerve regeneration as part of the primary study needed to be administered at 1 h post-crush, blood for the biomarker assay had to be drawn prior to treating the animals to prevent the treatments from obscuring the true effects of the optic nerve crush on biomarker concentrations. Although our findings demonstrate that GFAP, UCH-L1, and NFL concentrations are significantly elevated within a short time after injury, additional research is necessary to determine the kinetics.

These biomarkers could potentially be elevated in other clinical situations in which there is (1) interruption of structural integrity of the optic nerve or (2) presence of other pathologies affecting the optic nerve or possibly the retina (given the axons of the optic nerve originate from RGCs). Conceivably, increases in these plasma biomarkers that are a result of other optic nerve and/or retinal pathologies could have important clinical implications and need to be evaluated.

As discussed previously, the optic nerve is a white matter tract of the brain. The elevation of TBI plasma biomarkers in our model of TON supports the contention that TON is a focal form of TBI. A certain quandary exists, however, in that when there is concomitant damage to other portions of the brain beyond the optic nerve (a relatively common occurrence in TON), the elevation of plasma biomarkers cannot distinguish the regional source of the CNS damage. Nonetheless, well-designed human studies as well as additional animal studies may help determine if this assay is useful in both the diagnosis and management of TON in certain instances.

## References

[1] Andriessen, T.M., Horn, J., Franschman, G., van der Naalt, J., Haitsma, I., Jacobs, B., Steyerberg, E.W., and Vos, P.E. (2011). Epidemiology, severity classification, and outcome of moderate and severe traumatic brain injury: a prospective multicenter study. J. Neurotrauma 28, 2019–2031 21787177 10.1089/neu.2011.2034

[2] Levin, H.S., and Diaz-Arrastia, R.R. (2015). Diagnosis, prognosis, and clinical management of mild traumatic brain injury. Lancet Neurol. 14, 506–517 25801547 10.1016/S1474-4422(15)00002-2

[3] DeQuesada, I.M., 2^nd^, and Chokshi, F.H. (2014). Neuroimaging of acute traumatic brain injury: emphasis on magnetic resonance imaging and prognostic factors. Semin. Roentgenol. 49, 64–75 24342676 10.1053/j.ro.2013.10.003

[4] Lee, B. and Newberg, A. (2005). Neuroimaging in traumatic brain imaging. NeuroRx 2, 372–383 15897957 10.1602/neurorx.2.2.372 PMC1064998

[5] Amyot, F., Arciniegas, D.B., Brazaitis, M.P., Curley, K.C., Diaz-Arrastia, R., Gandjbakhche, A., Herscovitch, P., Hinds, S.R., 2nd, Manley, G.T., Pacifico, A., Razumovsky, A., Riley, J., Salzer, W., Shih, R., Smirniotopoulos, J.G., and Stocker, D. (2015). A review of the effectiveness of neuroimaging modalities for the detection of traumatic brain injury. J. Neurotrauma 32, 1693–1721 26176603 10.1089/neu.2013.3306 PMC4651019

[6] Bazarian, J.J., Biberthaler, P., Welch, R.D., Lewis, L.M., Barzo, P., Bogner-Flatz, V., Gunnar Brolinson, P., Buki, A., Chen, J.Y., Christenson, R.H., Hack, D., Huff, J.S., Johar, S., Jordan, J.D., Leidel, B.A., Lindner, T., Ludington, E., Okonkwo, D.O., Ornato, J., Peacock, W.F., Schmidt, K., Tyndall, J.A., Vossough, A., and Jagoda, A.S. (2018). Serum GFAP and UCH-L1 for prediction of absence of intracranial injuries on head CT (ALERT-TBI): a multicentre observational study. Lancet Neurol. 17, 782–789 30054151 10.1016/S1474-4422(18)30231-X

[7] Agoston, D.V., Shutes-David, A., and Peskind, E.R. (2017). Biofluid biomarkers of traumatic brain injury. Brain Inj. 31, 1195–1203 28981341 10.1080/02699052.2017.1357836

[8] Papa, L., Brophy, G.M., Welch, R.D., Lewis, L.M., Braga, C.F., Tan, C.N., Ameli, N.J., Lopez, M.A., Haeussler, C.A., Mendez Giordano, D.I., Silvestri, S., Giordano, P., Weber, K.D., Hill-Pryor, C., and Hack, D.C. (2016). Time course and diagnostic accuracy of glial and neuronal blood biomarkers GFAP and UCH-L1 in a large cohort of trauma patients with and without mild traumatic brain injury. JAMA Neurol. 73, 551–560 27018834 10.1001/jamaneurol.2016.0039 PMC8805143

[9] Mondello, S., Papa, L., Buki, A., Bullock, M.R., Czeiter, E., Tortella, F.C., Wang, K.K., and Hayes, R.L. (2011). Neuronal and glial markers are differently associated with computed tomography findings and outcome in patients with severe traumatic brain injury: a case control study. Crit. Care 15, R156 21702960 10.1186/cc10286 PMC3219030

[10] Diaz-Arrastia, R., Wang, K.K., Papa, L., Sorani, M.D., Yue, J.K., Puccio, A.M., McMahon, P.J., Inoue, T., Yuh, E.L., Lingsma, H.F., Maas, A.I., Valadka, A.B., Okonkwo, D.O., Manley, G.T. and TRACK-TBI Investigators (2014). Acute biomarkers of traumatic brain injury: relationship between plasma levels of ubiquitin C-terminal hydrolase-L1 and glial fibrillary acidic protein. J. Neurotrauma 31, 19–25 23865516 10.1089/neu.2013.3040 PMC3880090

[11] Childs, C., Martinez-Morillo, E., Wai, A.P., Zu, M.M., Diamandis, A. and Diamandis, E.P. (2013). Exploring the relationship between serum biomarkers, acute intracerebral changes and outcome after severe traumatic brain injury (TBI). Clin. Chem. Lab. Med. 51, e195–197 23612666 10.1515/cclm-2013-0216

[12] Rubenstein, R., Chang, B., Yue, J.K., Chiu, A., Winkler, E.A., Puccio, A.M., Diaz-Arrastia, R., Yuh, E.L., Mukherjee, P., Valadka, A.B., Gordon, W.A., Okonkwo, D.O., Davies, P., Agarwal, S., Lin, F., Sarkis, G., Yadikar, H., Yang, Z., Manley, G.T., Wang, K.K.W., the, T.-T.B.I.I., Cooper, S.R., Dams-O'Connor, K., Borrasso, A.J., Inoue, T., Maas, A.I.R., Menon, D.K., Schnyer, D.M., and Vassar, M.J. (2017). Comparing plasma phospho tau, total tau, and phospho tau-total tau ratio as acute and chronic traumatic brain injury biomarkers. JAMA Neurol. 74, 1063–1072 28738126 10.1001/jamaneurol.2017.0655 PMC5710183

[13] McMahon, P.J., Panczykowski, D.M., Yue, J.K., Puccio, A.M., Inoue, T., Sorani, M.D., Lingsma, H.F., Maas, A.I., Valadka, A.B., Yuh, E.L., Mukherjee, P., Manley, G.T., Okonkwo, D.O., and TRACK-TBI Investigators (2015). Measurement of the glial fibrillary acidic protein and its breakdown products GFAP-BDP biomarker for the detection of traumatic brain injury compared to computed tomography and magnetic resonance imaging. J. Neurotrauma 32, 527–533 25264814 10.1089/neu.2014.3635 PMC4394160

[14] Shahim, P., Gren, M., Liman, V., Andreasson, U., Norgren, N., Tegner, Y., Mattsson, N., Andreasen, N., Ost, M., Zetterberg, H., Nellgard, B., and Blennow, K. (2016). Serum neurofilament light protein predicts clinical outcome in traumatic brain injury. Sci. Rep. 6, 36791 27819296 10.1038/srep36791 PMC5098187

[15] Shahim, P., Zetterberg, H., Tegner, Y., and Blennow, K. (2017). Serum neurofilament light as a biomarker for mild traumatic brain injury in contact sports. Neurology 88, 1788–1794 28404801 10.1212/WNL.0000000000003912 PMC5419986

[16] Papa, L. (2016). Potential blood-based biomarkers for concussion. Sports Med. Arthrosc. Rev. 24, 108–115 27482776 10.1097/JSA.0000000000000117 PMC5055836

[17] Wang, K.K., Yang, Z., Zhu, T., Shi, Y., Rubenstein, R., Tyndall, J.A., and Manley, G.T. (2018). An update on diagnostic and prognostic biomarkers for traumatic brain injury. Expert Rev. Mol. Diagn. 18, 165–180 29338452 10.1080/14737159.2018.1428089 PMC6359936

[18] Welch, R.D., Ellis, M., Lewis, L.M., Ayaz, S.I., Mika, V.H., Millis, S., and Papa, L. (2017). Modeling the kinetics of serum glial fibrillary acidic protein, ubiquitin carboxyl-terminal hydrolase-L1, and S100B concentrations in patients with traumatic brain injury. J. Neurotrauma 34, 1957–1971 28031000 10.1089/neu.2016.4772 PMC6913786

[19] Meier, T.B., Nelson, L.D., Huber, D.L., Bazarian, J.J., Hayes, R.L., and McCrea, M.A. (2017). Prospective assessment of acute blood markers of brain injury in sport-related concussion. J. Neurotrauma 34, 3134–3142 28699381 10.1089/neu.2017.5046 PMC5678359

[20] Welch, R.D., Ayaz, S.I., Lewis, L.M., Unden, J., Chen, J.Y., Mika, V.H., Saville, B., Tyndall, J.A., Nash, M., Buki, A., Barzo, P., Hack, D., Tortella, F.C., Schmid, K., Hayes, R.L., Vossough, A., Sweriduk, S.T., and Bazarian, J.J. (2016). Ability of serum glial fibrillary acidic protein, ubiquitin C-terminal hydrolase-L1, and S100B to differentiate normal and abnormal head computed tomography findings in patients with suspected mild or moderate traumatic brain injury. J. Neurotrauma 33, 203–214 26467555 10.1089/neu.2015.4149 PMC4722555

[21] Mann, I. (1964). The Development of the Human Eye. Grune and Stratton. Inc.: New York

[22] Miller, N.R., Walsh, F.B., and Hoyt, W.F. (2005). *Walsh and Hoyt's Clinical Neuro-Ophthalmology,* Vol 1. Lippincott Williams & Wilkins

[23] Garman, R.H. (2011). Histology of the central nervous system. Toxicol. Pathol. 39, 22–35 21119051 10.1177/0192623310389621

[24] Tuttle, R., Braisted, J.E., Richards, L.J., and O'Leary, D. (1998). Retinal axon guidance by region-specific cues in diencephalon. Development 125, 791–801 9449662 10.1242/dev.125.5.791

[25] Ludwig, P.E., and Czyz, C.N. (2019). Embryology, eye malformations. In: *StatPearls*.Treasure Island, FL 29494102

[26] Tso, M.O., Shih, C.Y., and McLean, I.W. (1975). Is there a blood–brain barrier at the optic nerve head? Arch. Ophthalmol. 93, 815–825 828849 10.1001/archopht.1975.01010020703008

[27] Dowling, J.E. (1987). The Retina: An Approachable Part of the Brain. Harvard University Press: Cambridge

[28] Raz, N., and Levin, N. (2014). Cortical and white matter mapping in the visual system-more than meets the eye: on the importance of functional imaging to understand visual system pathologies. Front. Integr. Neurosci. 8, 68 25221482 10.3389/fnint.2014.00068 PMC4145715

[29] Hosseini, H.S., and Taber, L.A. (2018). How mechanical forces shape the developing eye. Prog. Biophys. Mol. Biol. 137, 25–36 29432780 10.1016/j.pbiomolbio.2018.01.004 PMC6085168

[30] Wilkinson, J.L. (2014). Neuroanatomy for Medical Students. Butterworth-Heinemann

[31] London, A., Benhar, I., and Schwartz, M. (2013). The retina as a window to the brain—from eye research to CNS disorders. Nat. Rev. Neurol. 9, 44 23165340 10.1038/nrneurol.2012.227

[32] Saggu, S.K., Chotaliya, H.P., Blumbergs, P.C., and Casson, R.J. (2010). Wallerian-like axonal degeneration in the optic nerve after excitotoxic retinal insult: an ultrastructural study. BMC Neurosci. 11, 97 20707883 10.1186/1471-2202-11-97 PMC2930628

[33] Kanamori, A., Catrinescu, M.M., Belisle, J.M., Costantino, S., and Levin, L.A. (2012). Retrograde and Wallerian axonal degeneration occur synchronously after retinal ganglion cell axotomy. Am. J. Pathol. 181, 62–73 22642911 10.1016/j.ajpath.2012.03.030 PMC3388161

[34] Harris, J.N. and Miller, N.R. (2017). Traumatic optic neuropathy, in: *Emergencies of the Orbit and Adnexa*. B. Mukherjee, and H. Yuen, H. (eds.). Springer India: New Delhi, pps. 113–137

[35] Lee, V., Ford, R.L., Xing, W., Bunce, C., and Foot, B. (2010). Surveillance of traumatic optic neuropathy in the UK. Eye (Lond.) 24, 240–250 19407847 10.1038/eye.2009.79

[36] Goldenberg-Cohen, N., Miller, N.R., and Repka, M.X. (2004). Traumatic optic neuropathy in children and adolescents. J. AAPOS 8, 20–27 14970795 10.1016/j.jaapos.2003.08.009

[37] Ford, R.L., Lee, V., Xing, W., and Bunce, C. (2012). A 2-year prospective surveillance of pediatric traumatic optic neuropathy in the United Kingdom. J. AAPOS 16, 413–417 23084375 10.1016/j.jaapos.2012.04.009

[38] Weichel, E.D., Colyer, M.H., Ludlow, S.E., Bower, K.S., and Eiseman, A.S. (2008). Combat ocular trauma visual outcomes during operations iraqi and enduring freedom. Ophthalmology 115, 2235–2245 19041478 10.1016/j.ophtha.2008.08.033

[39] Steinsapir, K.D., and Goldberg, R.A. (2011). Traumatic optic neuropathy: an evolving understanding. Am. J. Ophthalmol. 151, 928–933 e922. 21529765 10.1016/j.ajo.2011.02.007

[40] Levin, L.A., Beck, R.W., Joseph, M.P., Seiff, S., and Kraker, R. (1999). The treatment of traumatic optic neuropathy: the International Optic Nerve Trauma Study. Ophthalmology 106, 1268–1277 10406604 10.1016/s0161-6420(99)00707-1

[41] Rajiniganth, M.G., Gupta, A.K., Gupta, A., and Bapuraj, J.R. (2003). Traumatic optic neuropathy: visual outcome following combined therapy protocol. Arch. Otolaryngol. Head Neck Surg. 129, 1203–1206 14623751 10.1001/archotol.129.11.1203

[42] Kurimoto, T., Yin, Y., Omura, K., Gilbert, H.Y., Kim, D., Cen, L.P., Moko, L., Kugler, S., and Benowitz, L.I. (2010). Long-distance axon regeneration in the mature optic nerve: contributions of oncomodulin, cAMP, and pten gene deletion. J. Neurosci. 30, 15654–15663 21084621 10.1523/JNEUROSCI.4340-10.2010 PMC3001271

[43] Li, Y., Andereggen, L., Yuki, K., Omura, K., Yin, Y., Gilbert, H.Y., Erdogan, B., Asdourian, M.S., Shrock, C., de Lima, S., Apfel, U.P., Zhuo, Y., Hershfinkel, M., Lippard, S.J., Rosenberg, P.A., and Benowitz, L. (2017). Mobile zinc increases rapidly in the retina after optic nerve injury and regulates ganglion cell survival and optic nerve regeneration. Proc. Natl. Acad. Sci. U. S. A. 114, E209–E218 28049831 10.1073/pnas.1616811114 PMC5240690

[44] Trakhtenberg, E.F., Li, Y., Feng, Q., Tso, J., Rosenberg, P.A., Goldberg, J.L., and Benowitz, L.I. (2018). Zinc chelation and Klf9 knockdown cooperatively promote axon regeneration after optic nerve injury. Exp. Neurol. 300, 22–29 29106981 10.1016/j.expneurol.2017.10.025 PMC5745290

[45] Holt, D., Por, E., Cleland, J., Harris, J., Gorantla, V., Sandoval, M., Thomas-Benson, C., Harris, L., Negaard, A., and Benowitz, L.I. (2018). Development of a porcine optic nerve injury model. Invest. Ophthalmol. Vis. Sci. 59, 314–314

[46] Bramblett, G., Harris, J., Cleland, J., Gorantla, V., Sandoval, M., Harris, L., Edsall, P., Benowitz, L., Goldberg, J.L., and Holt, A. (2019). Optimization of crush force in a porcine model of traumatic optic neuropathy. Invest. Ophthalmol. Vis. Sci. 60, 2263–226331112611

